# Evaluation of the Fermentation Characteristics of Prebiotic‐Containing Granola and Short‐Chain Fatty Acid Production in an In Vitro Gut Microbiota Model

**DOI:** 10.1002/fsn3.70252

**Published:** 2025-05-07

**Authors:** Hiroyuki Sasaki, Hirofumi Masutomi, Yota Kobayashi, Kiyotsuna Toyohara, Keiichiro Imaizumi, Yasunori Nakayama, Katsuyuki Ishihara

**Affiliations:** ^1^ Research & Development Division Calbee, Inc. Tochigi Japan; ^2^ Healthcare New Business Division Teijin Limited Tokyo Japan

**Keywords:** granola, gut microbiota model, prebiotics, short‐chain fatty acid

## Abstract

Mammalian gut microbiota is essential for host metabolism and immune function and produces short‐chain fatty acids (SCFAs) via fermentation of dietary fibers and prebiotic materials. In this study, we aimed to investigate the effects of prebiotic‐containing granola on SCFA production and gut microbiota composition using an in vitro pig feces anaerobic fermentation model. Six prebiotic materials (inulin, resistant starch, fructooligosaccharides, galactooligosaccharides, cacao mass, and barley) were added to granola, and their fermentation characteristics were evaluated. SCFA analysis revealed that prebiotic materials significantly increased the total SCFA production after 48 h, with inulin, fructooligosaccharide, and galactooligosaccharide showing the highest production. Bacterial growth was enhanced in multiple genera; however, the growth varied with different prebiotics. Previous studies have suggested the presence of responders and non‐responders to individual prebiotics, emphasizing the need to combine diverse prebiotics to overcome such variations. Prebiotic‐containing granola, but not prebiotic material, significantly increased the abundances of *Blautia* and *Prevotella* bacteria. Additionally, prebiotic‐containing granola significantly increased the abundance of all bacteria more than granola alone. Base granola only exhibited high fermentability, whereas prebiotic‐containing granola modulated the gut microbiota populations and enhanced SCFA production. These findings highlight the benefits of combining prebiotics with granola, suggesting that dietary interventions alter the gut microbiota composition and metabolism. Our results provide valuable insights and can contribute to future human trials of the investigated compounds.

## Graphical abstract

This study explored the effects of prebiotic‐containing granola on short‐chain fatty acid (SCFA) production and gut microbiota composition using an in vitro pig feces anaerobic fermentation model. The results showed that prebiotic‐containing granola significantly enhanced SCFA production and modulated the gut microbiota, particularly increasing the abundance of Blautia and Prevotella, indicating potential benefits of prebiotics in dietary interventions for gut health.

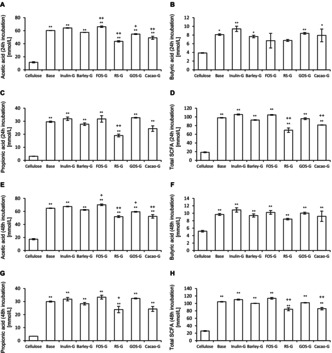

## 1 Introduction

The mammalian gut microbiota consists of approximately 100 trillion bacteria. These microbes are involved in various physiological processes, including nutrient absorption and host metabolism (Marchesi et al. 2016). Short‐chain fatty acids (SCFAs), produced by the gut microbiota, play a regulatory role in host physiology and influence multiple functions, including gut immunity, metabolism, and the endocrine and nervous systems. Altered SCFA levels have been associated with conditions such as obesity, hypertension, and disorders of the central nervous system (Chambers et al. 2018; Lange et al. 2023; Overby and Ferguson 2021; Silva et al. 2020).

SCFAs are produced through the fermentation of indigestible food components, such as dietary fiber and oligosaccharides, by the gut microbiota (Koh et al. 2016). Upon reaching the large intestine, dietary fibers are broken down into monosaccharides by enzymes produced by intestinal bacteria. These monosaccharides are generally converted into pyruvate via the glycolytic pathway, and SCFAs are subsequently synthesized from pyruvate through distinct metabolic routes (Koh et al. 2016). Indigestible components play a key role in SCFA production. Moreover, indigestible and fermentable food components—collectively referred to as “prebiotics”—significantly influence both the composition of the gut microbiota and SCFA production (David et al. 2014). Inulin, a well‐known prebiotic, alters the gut microbiota composition and increases the abundance of butyric acid‐producing bacteria (Barber et al. 2022; Valcheva et al. 2015). Elevated levels of butyric acid have been shown to enhance insulin resistance in the liver, as well as increase blood lipid levels and hepatic lipid accumulation (Hu et al. 2020).

Granola, a food composed of a mixture of grains such as oats and rye flour, is rich in dietary fiber. We previously reported that consuming granola at breakfast promotes bowel movements in adult women and primary school children (Kyo et al. 2017; Matsumoto et al. 2023). In addition, granola intake has been shown to lower blood pressure in patients undergoing hemodialysis, reduce levels of the urinary toxin indoxyl sulfate, and increase gut microbial diversity (Nagasawa et al. 2024, 2021). These findings suggest that granola consumption may also promote SCFA production. A previous study identified two groups: responders, in whom granola consumption led to changes in gut microbiota composition and increased SCFA production, and non‐responders, in whom SCFA levels remained unchanged (Yamauchi et al. 2023). These differences between responders and non‐responders are likely due to individual variations in gut microbiota composition.

In addition to granola, many prebiotic compounds enhance SCFA production; however, both responders and non‐responders may exist for each type of prebiotic. Even when the same prebiotic is consumed, differences in gut microbiota composition among individuals can lead to varying levels of SCFA production. For example, barley improves glucose metabolism and lowers blood glucose levels in individuals with a high relative abundance of *Prevotella* in their gut (Kovatcheva‐Datchary et al. 2015). Similarly, inulin significantly alters gut microbiota composition in individuals who habitually consume high amounts of dietary fiber and exhibit a high relative abundance of *Bifidobacterium* (Healey et al. 2018). In a previous study involving healthy adults from six regions in Asia (Inner Mongolia, Xinjiang, and Gansu in China; Mongolia; Singapore; and Indonesia), probiotic administration resulted in region‐specific changes in gut microbiota composition, suggesting that differences in gut enterotypes may influence the effects of probiotics (Hou et al. 2020). Therefore, substantial individual variability in the effects of prebiotics on gut microbiota composition and SCFA production should be considered in clinical practice. Given the existence of both responders and non‐responders to granola and other prebiotics, combining granola with specific prebiotic materials tailored to individual gut microbiota profiles may be an effective strategy. However, the combined effects of granola and various prebiotics on SCFA production and gut microbiota composition have not been systematically evaluated. This study is novel in that it investigates the fermentation characteristics and microbiota‐modulating effects of granola supplemented with different prebiotics using an in vitro gut microbiota model. To this end, we prepared a granola formulation based on commercially available products, supplemented it with various prebiotic materials, and assessed its fermentation properties using an in vitro gut microbiota model to elucidate its effects on gut microbiota composition and SCFA production.

## 2 Materials and Methods

### 2.1 Prebiotic Materials and Prebiotic‐Containing Granola

The following prebiotics were used in this study: inulin, resistant starch (RS), fructooligosaccharide (FOS), galactooligosaccharide (GOS), cacao mass, and barley (all obtained from Calbee Inc., Tokyo, Japan). Prebiotic‐containing granolas were prepared by mixing each prebiotic with fruit granola (Calbee Inc.), which served as the base. The resulting formulations were named as follows: inulin‐G, RS‐G, FOS‐G, GOS‐G, cacao‐G, and barley‐G. Their nutritional compositions are shown in Table 1, and detailed ingredient information is provided in Table S1. Cellulose was used as a control.

**TABLE 1:** Nutritional composition of each prebiotic‐containing granola (/100 g).

	Base	Inulin‐G	Barley‐G	FOS‐G	RS‐G	GOS‐G	Cacao‐G
Energy (kcal)	443.8	366.7	383.3	366.7	416.7	350.0	433.3
Protein (g)	6.9	6.7	6.7	6.7	5.0	6.7	10.0
Fat (g)	15.9	5.0	5.0	5.0	11.7	3.3	11.7
Carbohydrate (g)	72.5	83.4	83.3	83.3	80.0	86.6	73.4
Sugar (g)	63.4	61.7	73.3	60.0	66.7	63.3	66.7
Fiber (g)	9.1	21.7	10	23.3	13.3	23.3	6.7
Sodium content (g)	0.6	0.5	0.5	0.7	0.7	0.3	0.7

Abbreviations: Barley‐G, barley‐containing granola; Base, base granola; Cacao‐G, cacao mass‐containing granola; FOS‐G, fructooligosaccharide‐containing granola; GOS, galactooligosaccharide‐containing granola; Inulin‐G, inulin‐containing granola; RS‐G, resistant starch‐containing granola.

Certain gut bacteria are known to metabolize prebiotics and promote SCFA production (Abell et al. 2008; Aoki et al. 2021; Chambers et al. 2019; Fehlbaum et al. 2018; Kovatcheva‐Datchary et al. 2015; Liu et al. 2017; Scott et al. 2014; Shin et al. 2022; Tochio et al. 2018; Tzounis et al. 2008; Ze et al. 2012). These bacteria were selected as target species to evaluate the effects of each prebiotic material (see Table 2).

**TABLE 2:** Target bacteria for each prebiotic material.

Prebiotic materials	Target bacteria	Reference
Inulin	*Bacteroides*	Aoki et al. (2021) and Chambers et al. (2019)
Barley	*Prevotella*	Fehlbaum et al. (2018) and Kovatcheva‐Datchary et al. (2015)
FOS	*Faecalibacterium*	Scott et al. (2014) and Tochio et al. (2018)
RS	*Ruminococcus*	Abell et al. (2008) and Ze et al. (2012)
GOS	*Bifidobacterium*	Fehlbaum et al. (2018) and Liu et al. (2017)
Cacao mass	*Blautia*	Shin et al. (2022) and Tzounis et al. (2008)

Abbreviations: FOS, fructooligosaccharide; GOS, galactooligosaccharide; RS, resistant starch.

### 2.2 Preparation of Indigestible Components

To mimic in vivo digestion and simulate the colonic environment, the indigestible components of cacao mass, barley, and granola samples were prepared through enzymatic digestion.

First, the samples were ground using a blender and passed through a 1‐mm mesh sieve (Sanpo Inc., Saitama, Japan). Then, 20 g of each sample was mixed with 300 mL of petroleum ether and stirred for 5 min to remove lipids. The mixture was filtered through a 0.2‐μm membrane, and the residue was air‐dried to eliminate volatile compounds.

Next, 10 g of the dried residue was combined with 50 mL of hot water and heated in a boiling water bath for 5 min. After cooling, artificial gastric fluid (2 g NaCl and 7 mL HCl in 1 L of water) and 75 mg of pepsin were added. The mixture was incubated at 37°C for 2 h, and the pH was adjusted to 7.0 using 12 N NaOH.

Subsequently, artificial intestinal fluid (1.42 g Na_2_HPO_4_, 1.36 g KH_2_PO_4_, and 0.58 g NaCl in 1 L of water), 25 mg of pancreatin, and 1000 mg of bile powder were added. The mixture was incubated at 37°C for 3 h. Then, 200 mL of ethanol was added, and the solution was centrifuged at 8000 rpm for 5 min. The supernatant was discarded, and the residue was washed with 50% ethanol and acetone, followed by filtration and overnight drying in a vacuum desiccator.

### 2.3 In Vitro Colonic Fermentation

In vitro colonic fermentation was conducted based on a previously described method (Kilua et al. 2019), with slight modifications. Fecal samples were collected directly from the rectum of four antibiotic‐free Mangalica pigs (approximately 6 months old) raised at the Tokachi Royal Mangalica Farm (Marukatsu Co. Ltd., Hokkaido, Japan). The feces were diluted to 20% (w/v) in 0.85% saline solution.

The fecal slurry was added to pH‐controlled anaerobic fermenters (Bio Jr.8; ABLE Corp., Tokyo, Japan) at a final concentration of 2.2% (w/v) of the total culture volume. Nutrient broth (Difco, Sparks, MD, USA), prebiotic samples, and sterile water were subsequently added. The final concentrations in the medium were 0.8% nutrient broth, 1.5% sample, and 1.7% pig feces (w/v).

Cultures were incubated for 48 h under anaerobic conditions, with the pH maintained above 5.50. Culture samples (1 mL) were collected at 0, 24, and 48 h and stored at −80°C for subsequent DNA extraction and SCFA analysis.

### 2.4 SCFA Analysis

Short‐chain fatty acids (SCFAs), including acetate, propionate, and butyrate, were quantified using the Prominence Organic Acid Analysis System (Shimadzu Corp., Kyoto, Japan). Separation was performed with a Shim‐pack SCR‐102H column (8.0 × 300 mm, 7 μm) and a guard column of the same material (6.0 × 50 mm, 7 μm). The mobile phase consisted of a 5.0 mmol/L aqueous solution of p‐toluenesulfonic acid. The pH buffer reagent contained 5.0 mmol/L p‐toluenesulfonic acid, 20 mM Bis‐Tris, and 0.1 mM EDTA. The flow rate was set to 0.8 mL/min at 40°C, and detection was performed via conductivity.

Sample preparation was based on a previously reported method (Han et al. 2016), with slight modifications. Culture media were centrifuged at 10,000 × g for 5 min at 4°C. Then, 200 μL of 0.5 M perchloric acid was added to 90 μL of the supernatant and vortexed. The mixture was centrifuged again at 10,000 × g for 10 min at 4°C, and the resulting supernatant was used for HPLC analysis.

### 2.5 Bacterial DNA Extraction and Quantitative Polymerase Chain Reaction (qPCR)

Genomic DNA was extracted from the culture media using the NucleoSpin DNA Stool Kit (Macherey‐Nagel, Düren, Germany), following the manufacturer's protocol. DNA concentrations were measured using the Multiskan SkyHigh Microplate Spectrophotometer with μDrop Plates (Thermo Fisher Scientific, Waltham, MA, USA), and samples were stored at −80°C.

qPCR was performed using the Fast SYBR Green Master Mix (Thermo Fisher Scientific) and the CFX Opus 384 Real‐Time PCR Detection System (Bio‐Rad Laboratories Inc., Hercules, CA, USA). Primer sequences are listed in Table 3. PCR conditions were as follows: initial denaturation at 95°C for 20 s, followed by 40 cycles of 95°C for 3 s and 60°C for 30 s. Ct values were determined according to the manufacturer's instructions.

**TABLE 3:** List of primers used for real‐time quantitative polymerase chain reaction (qPCR) analysis.

Target	Primer sequence	Annealing[°C]
All bacteria	5′‐AGAGTTTGATCATGGCTCAG‐3′	60
	5′‐ACCGCGACTGCTGCTGGCAC‐3′	
*Bacteroides*	5′‐GAGAGGAAGGTCCCCCAC‐3′	60
	5′‐CGCTACTTGGCTGGTTCAG‐3′	
*Prevotella*	5′‐CACRGTAAACGATGGATGCC‐3′	60
	5′‐GGTCGGGTTGCAGACC‐3′	
*Faecalibacterium*	5′‐CTAACTACGTGCCAGCAGCC‐3′	60
	5′‐GCCTTCGCCACTGGTGTTCC‐3′	
*Ruminococcus*	5′‐GAAAGCGTGGGGAGCAAACAGG‐3′	60
	5′‐GACGACAACCATGCACCACCTG‐3′	
*Bifidobacterium*	5′‐CTCCTGGAAACGGGTGG‐3′	60
	5′‐GGTGTTCTTCCCGATATCTACA‐3′	
*Blautia*	5′‐CGGTACCTGACTAAGAAGC‐3′	60
	5′‐GTTCCTCCTAATATCTACGC‐3′	

The relative abundances of *Prevotella*, *Faecalibacterium*, and *Ruminococcus* were calculated using standard curves generated from pooled DNA extracted from all experimental groups after 48 h of incubation. For *Bacteroides*, *Bifidobacterium*, and *Blautia*, standard DNA solutions were prepared from cultured strains and serially diluted. The details are as follows: *Bacteroides*: In‐house strain; DNA extracted using the SimplePrep kit (Takara Bio, Gunma, Japan). *Bifidobacterium*: JCM 1192ᵀ strain; DNA extracted using the NucleoSpin kit. *Blautia*: JCM 1395ᵀ strain; DNA extracted using the SimplePrep kit.

All procedures were performed in accordance with the respective kit protocols.

### 2.6 Statistical Analyses

All data are presented as the mean ± standard error of the mean (SEM). Statistical analyses were performed using GraphPad Prism version 9.5.1 (GraphPad Software Inc., San Diego, CA, USA). The Shapiro–Wilk test was used to assess the normality of the data. If the data followed a normal distribution, one‐way ANOVA followed by Dunnett's post hoc test was applied. For non‐normally distributed data, the Kruskal–Wallis test followed by Dunn's post hoc test was used. A *p*‐value of < 0.05 was considered statistically significant.

## 3 Results

### 3.1 SCFA Production and Modulation of Gut Microbiota by Prebiotic Fermentation

**FIGURE 1:**
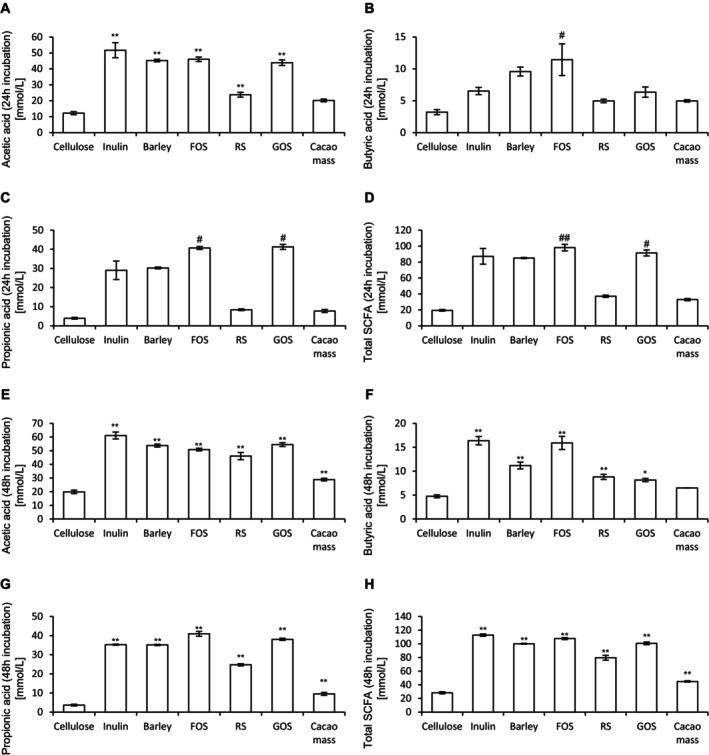
Cultivation of prebiotic materials in an in vitro microbiota model produces short‐chain fatty acids (SCFA). Levels of SCFAs ((A) and (E), acetic acid, (B) and (F) butyric acid, (C) and (G) propionic acid, and (D) and (H) total SCFAs) after 24 h (A)–(D) and 48 h (E)–(H) of incubation of different prebiotic materials. All values are represented as the mean ± standard error of the mean (SEM; *n* = 3 for all groups). **p < 0.01 and *p < 0.05 vs. cellulose, analyzed via one‐way analysis of variance (ANOVA), followed by Dunnett's post hoc test. ##*p* < 0.01 and #*p* < 0.05 vs. cellulose, analyzed via the Kruskal–Wallis test, followed by Dunn's post hoc test. FOS, fructooligosaccharide; RS, resistant starch; GOS, galactooligosaccharide.

We investigated whether SCFA production and gut microbiota composition varied depending on the type of prebiotic material, using six different prebiotics in an in vitro fermentation system with pig feces. After 24 h of incubation, acetic acid concentrations significantly increased with all prebiotic materials except cacao mass. Butyric acid concentrations increased significantly only with FOS, while propionic acid concentrations increased significantly with both FOS and GOS. Furthermore, FOS and GOS significantly elevated total SCFA levels—the sum of acetic, butyric, and propionic acid concentrations—compared to cellulose (Figure 1A–D). After 48 h of incubation, both acetic and propionic acid concentrations were significantly higher for all prebiotic materials compared to cellulose. Butyric acid concentrations also significantly increased with all prebiotics except cacao mass. Notably, all prebiotic treatments led to a greater increase in total SCFA concentrations than cellulose (Figure 1E–H). These results indicate that supplementation with prebiotic materials, combined with 48‐h incubation, enhanced SCFA production in the in vitro fermentation system.

**FIGURE 2:**
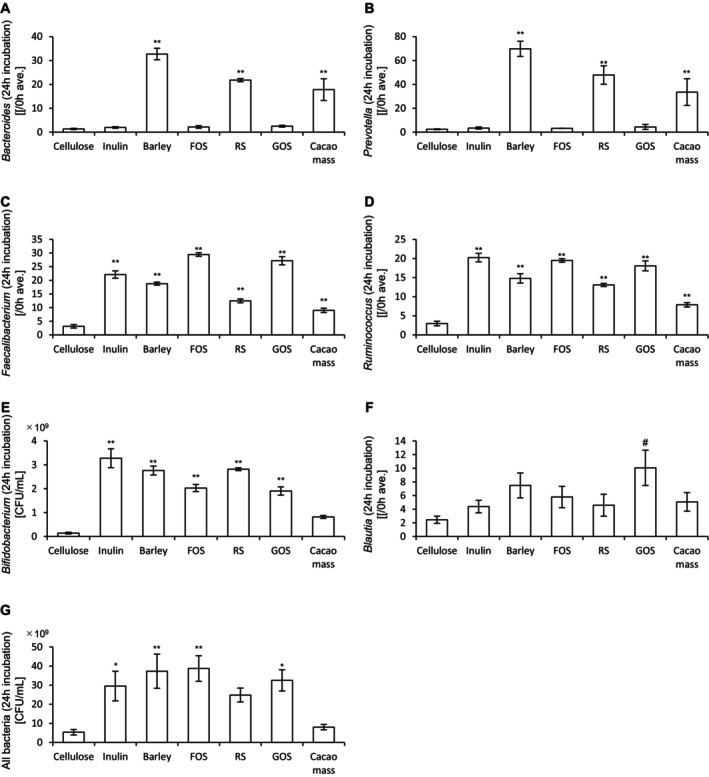
Cultivation of prebiotic materials in an in vitro gut microbiota model for 24 h increases the bacterial abundance. (A)–(G) Abundances of (A) *Bacteroides*, (B) *Prevotella*, (C) *Faecalibacterium*, (D) *Ruminococcus*, (E) *Bifidobacterium*, (F) *Blautia*, and (G) all bacteria after 24‐h incubation with different prebiotic materials. All values are represented as the mean ± SEM (*n* = 3 for all groups). **p < 0.01 and *p < 0.05, vs. cellulose, analyzed via one‐way ANOVA, followed by Dunnett's post hoc test. #*p* < 0.05 vs. cellulose, analyzed via the Kruskal–Wallis test, followed by Dunn's post hoc test. FOS, fructooligosaccharide; GOS, galactooligosaccharide; RS, resistant starch.

**FIGURE 3:**
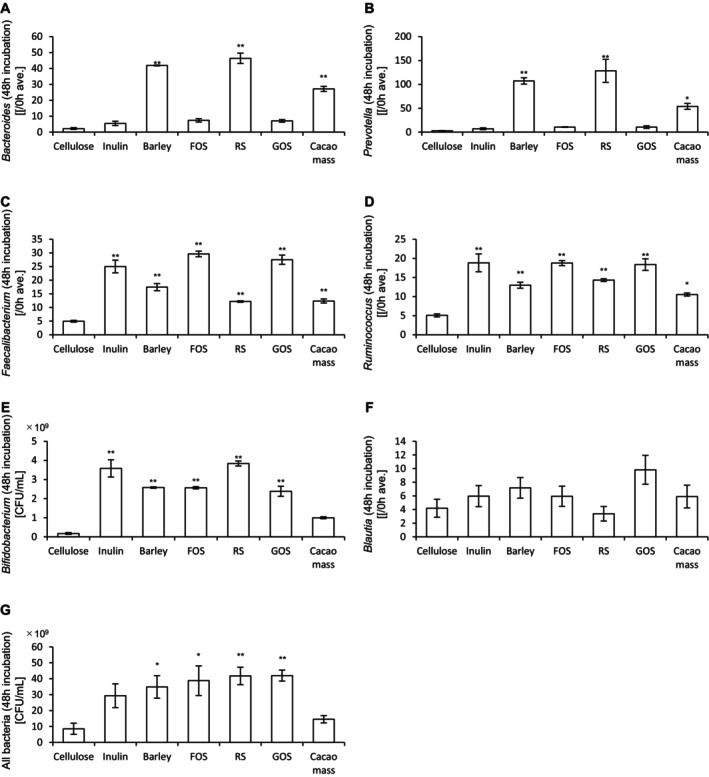
Cultivation of prebiotic materials in an in vitro gut microbiota model for 48 h increases the bacterial abundance. (A)–(G) Abundances of (A) *Bacteroides*, (B) *Prevotella*, (C) *Faecalibacterium*, (D) *Ruminococcus*, (E) *Bifidobacterium*, (F) *Blautia*, and (G) all bacteria after 48‐h incubation of different prebiotic materials. All values are represented as the mean ± SEM (*n* = 3 for all groups). **p < 0.01 and *p < 0.05 vs. cellulose, analyzed via one‐way ANOVA, followed by Dunnett's post hoc test. #*p* < 0.05 vs. cellulose, analyzed via the Kruskal–Wallis test, followed by Dunn's post hoc test. FOS, fructooligosaccharide; RS, resistant starch; GOS, galactooligosaccharide.

Next, the abundance of bacteria in the culture medium was measured. In addition, target bacterial species for each prebiotic material were evaluated. The results of 24‐h and 48‐h incubations are presented in Figures 2 and 3, respectively. After 24 h of incubation, barley, RS, and cacao mass significantly increased the abundances of *Bacteroides* and *Prevotella* compared to cellulose. All prebiotic materials significantly increased the abundances of *Faecalibacterium* and *Ruminococcus*. Notably, the abundance of *Bifidobacterium* was significantly increased by all prebiotics except cacao mass. In contrast, *Blautia* abundance was significantly increased only by GOS. The overall bacterial abundance was significantly increased by all prebiotics, except RS and cacao mass. After 48 h of incubation, barley, RS, and cacao mass again significantly increased the abundances of *Bacteroides* and *Prevotella* relative to cellulose. All prebiotics increased the abundances of *Faecalibacterium* and *Ruminococcus*, while *Bifidobacterium* abundance was significantly increased by all prebiotics except cacao mass. However, no significant increase in *Blautia* abundance was observed. The overall bacterial abundance was significantly increased by all prebiotics, except inulin and cacao mass.

Collectively, these results suggest that prebiotic materials increase the abundances of various bacterial species, including both target and non‐target taxa, and that the specific bacterial responses depend on the type of prebiotic used.

### 3.2 SCFA Production and Microbial Responses to Prebiotic‐Containing Granola

SCFA production was enhanced by the anaerobic in vitro fermentation of each prebiotic material. However, in addition to increases in target bacterial populations, the abundances of various non‐target bacteria also increased. Therefore, we prepared granola mixtures supplemented with each prebiotic material and analyzed their effects in comparison to granola fermented alone.

**FIGURE 4:**
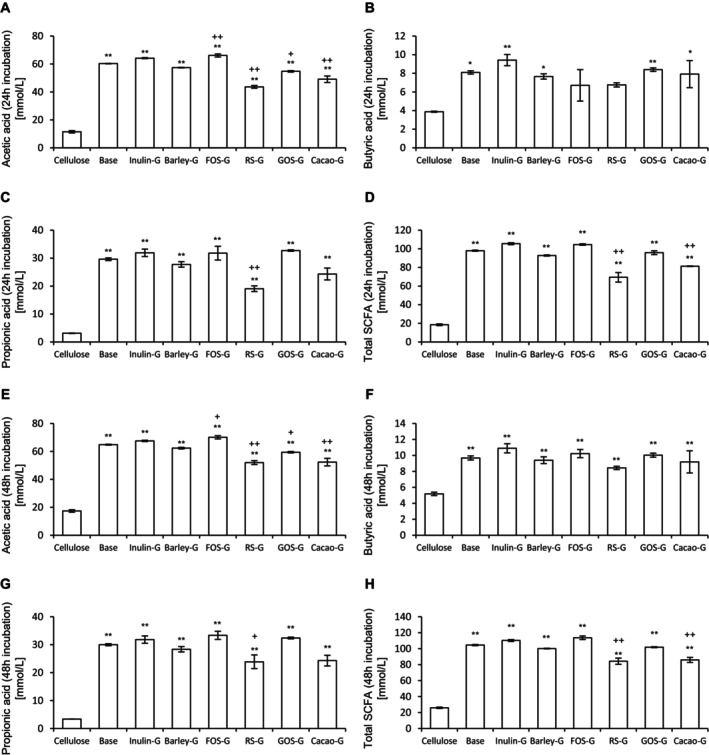
Cultivation of prebiotic‐containing granola in an in vitro microbiota model produces SCFAs. Levels of SCFAs ((A) and (E), acetic acid, (B) and (F) butyric acid, (C) and (G) propionic acid, and (D) and (H) total SCFAs) after 24 h (A)–(D) and 48 h (E)–(H) of incubation of prebiotic‐containing granola. All values are represented as the mean ± SEM (*n* = 3 for all groups). **p < 0.01 and *p < 0.05 vs. cellulose, analyzed via one‐way ANOVA, followed by Dunnett's post hoc test. ++*p* < 0.01 and + *p* < 0.05 vs. base, analyzed via one‐way analysis ANOVA, followed by Dunnett's post hoc test. ##*p* < 0.01 and #*p* < 0.05 vs. cellulose, analyzed via the Kruskal–Wallis test, followed by Dunn's post hoc test. Barley‐G, barley‐containing granola; Base, base granola; Cacao‐G, cacao mass‐containing granola; FOS‐G, fructooligosaccharide‐containing granola; GOS, galactooligosaccharide‐containing granola; Inulin‐G, inulin‐containing granola; RS‐G, resistant starch‐containing granola.

SCFA analysis results are shown in Figure 4. After 24 h of incubation, the production of acetic acid, propionic acid, and total SCFAs was significantly higher in all granola groups compared to the cellulose group. Butyric acid production was significantly increased in the base (*p* = 0.0088), inulin‐G (*p* = 0.0007), barley‐G (*p* = 0.02), GOS‐G (*p* = 0.005), and cacao‐G (*p* = 0.0123) groups compared to cellulose. No significant differences in SCFA production were observed between the base granola and the prebiotic‐supplemented granola groups. However, compared to the base, FOS‐G significantly increased acetic acid levels (*p* = 0.0077), whereas RS‐G (*p* = 0.0001), GOS‐G (*p* = 0.0117), and cacao‐G (*p* = 0.0001) significantly decreased acetic acid production. RS‐G (*p* = 0.0008 and *p* = 0.0001) and cacao‐G (*p* = 0.091 and *p* = 0.0012) significantly reduced propionic acid and total SCFA levels, respectively, compared to the base. After 48 h of incubation, the concentrations of acetic acid, butyric acid, propionic acid, and total SCFAs were significantly increased in all prebiotic‐containing granola groups compared to cellulose. Consistent with the 24‐h results, FOS‐G produced significantly more acetic acid than the base (*p* = 0.0493). In contrast, RS‐G (*p* = 0.0001 and *p* = 0.0001 for acetic acid and total SCFAs), GOS‐G (*p* = 0.044 for acetic acid), and cacao‐G (*p* = 0.0001 and *p* = 0.0002 for acetic acid and total SCFAs, respectively) showed significantly reduced levels compared to the base. Moreover, RS‐G significantly decreased propionic acid production compared to the base (*p* = 0.0429). Notably, no significant differences were found between the base and any prebiotic‐containing granola group in overall SCFA production. These results suggest that while prebiotic‐containing granola enhances SCFA production compared to cellulose, it does not necessarily result in greater SCFA production than granola alone.

**FIGURE 5:**
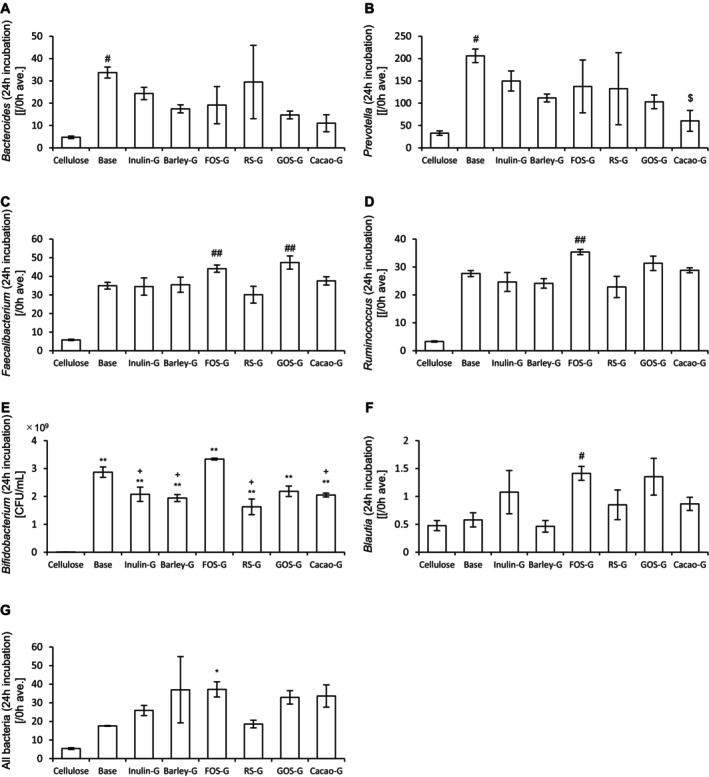
Cultivation of prebiotic‐containing granola in an in vitro gut microbiota model for 24 h increases the bacterial abundance. (A)–(G) Abundances of (A) *Bacteroides*, (B) *Prevotella*, (C) *Faecalibacterium*, (D) *Ruminococcus*, (E) *Bifidobacterium*, (F) *Blautia*, and (G) all bacteria after 24‐h incubation of prebiotic‐containing granola. All values are represented as the mean ± SEM (*n* = 3 for all groups). **p < 0.01 and *p < 0.05 vs. cellulose, analyzed via one‐way ANOVA, followed by Dunnett's post hoc test. +*p* < 0.05 vs. base, analyzed via one‐way ANOVA, followed by Dunnett's post hoc test. #*p* < 0.05 vs. cellulose, analyzed via the Kruskal–Wallis test, followed by Dunn's post hoc test. $*p* < 0.05 vs. base, analyzed via the Kruskal–Wallis test, followed by Dunn's post hoc test. Barley‐G, barley‐containing granola; Base, base granola; Cacao‐G, cacao mass‐containing granola; FOS‐G, fructooligosaccharide‐containing granola; GOS, galactooligosaccharide‐containing granola; Inulin‐G, inulin‐containing granola; RS‐G, resistant starch‐containing granola.

**FIGURE 6:**
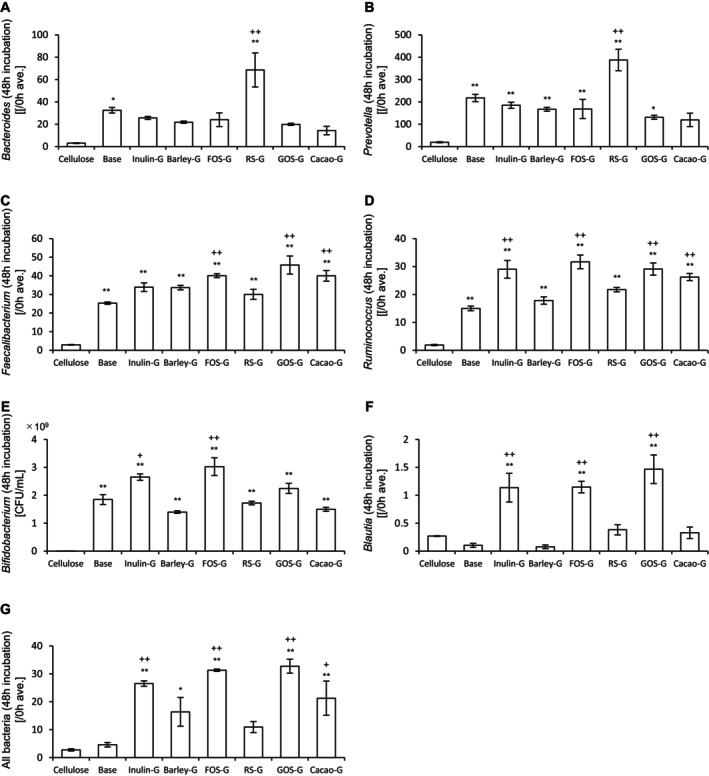
Cultivation of prebiotic‐containing granola in an in vitro gut microbiota model for 48 h increases bacterial abundance. (A)–(G) Abundances of (A) *Bacteroides*, (B) *Prevotella*, (C) *Faecalibacterium*, (D) *Ruminococcus*, (E) *Bifidobacterium*, (F) *Blautia*, and (G) all bacteria after 48‐h incubation of prebiotic‐containing granola. All values are represented as the mean ± SEM (*n* = 3 for all groups). **p < 0.01 and *p < 0.05 vs. cellulose, analyzed via one‐way ANOVA, followed by Dunnett's post hoc test. ++*p* < 0.01 and + *p* < 0.05 vs. base, analyzed via one‐way ANOVA, followed by Dunnett's post hoc test. Barley‐G, barley‐containing granola; Base, base granola; Cacao‐G, cacao mass‐containing granola; FOS‐G, fructooligosaccharide‐containing granola; GOS, galactooligosaccharide‐containing granola; Inulin‐G, inulin‐containing granola; RS‐G, resistant starch‐containing granola.

Bacterial abundance was also measured. Results after 24 and 48 h of incubation are shown in Figures 5 and 6, respectively. After 24 h of incubation, the abundances of *Bacteroides* (*p* = 0.0193) and *Prevotella* (*p* = 0.0027) were significantly higher in the base group than in the cellulose group. *Faecalibacterium* abundance was significantly increased in the FOS‐G (*p* = 0.0318) and GOS‐G (*p* = 0.0095) groups, while *Ruminococcus* abundance was significantly higher in the FOS‐G group (*p* = 0.0095) compared to cellulose. Notably, *Bifidobacterium* abundance was significantly increased in all granola groups relative to the cellulose group. *Blautia* abundance was also significantly higher in the FOS‐G group (*p* = 0.0442) compared to cellulose. Among all groups, the FOS‐G group showed significant increases in the abundances of all evaluated bacterial species relative to the cellulose group. Interestingly, none of the prebiotic‐containing granola samples significantly increased the abundance of gut bacteria during this period. In contrast, cacao‐G significantly decreased *Prevotella* abundance (*p* = 0.0419), and inulin‐G (*p* = 0.0383), barley‐G (*p* = 0.0145), RS‐G (*p* = 0.0014), and cacao‐G (*p* = 0.0316) significantly reduced *Bifidobacterium* abundance. After 48 h of incubation, the abundances of *Faecalibacterium*, *Ruminococcus*, and *Bifidobacterium* were significantly increased in all granola groups compared to the cellulose group. *Prevotella* abundance was significantly higher in all granola groups except cacao‐G (*p* = 0.0809). *Bacteroides* abundance was significantly higher in the base and RS‐G groups (*p* = 0.0001) than in the cellulose group. *Blautia* abundance was significantly elevated in the inulin‐G (*p* = 0.0033), FOS‐G (*p* = 0.003), and GOS‐G (*p* = 0.0002) groups. Furthermore, the total bacterial abundance was significantly increased in the inulin‐G (*p* = 0.0003), barley‐G (*p* = 0.0338), FOS‐G (*p* = 0.0001), GOS‐G (*p* = 0.0001), and cacao‐G (*p* = 0.0035) groups compared to cellulose. After 48 h of incubation, the abundances of all evaluated bacterial taxa were significantly higher in all prebiotic‐containing granola groups compared to the base group. Specifically, RS‐G significantly increased the abundances of *Bacteroides* (*p* = 0.0072) and *Prevotella* (*p* = 0.004). *Faecalibacterium* abundance was significantly increased by FOS‐G (*p* = 0.0084), GOS‐G (*p* = 0.0004), and cacao‐G (*p* = 0.0087). *Ruminococcus* abundance was significantly increased by inulin‐G (*p* = 0.0007), FOS‐G (*p* = 0.0001), GOS‐G (*p* = 0.0007), and cacao‐G (*p* = 0.0047). For *Bifidobacterium*, significant increases were observed in the inulin‐G (*p* = 0.0182) and RS‐G (*p* = 0.0009) compared to the base. *Blautia* abundance was significantly higher in the inulin‐G (*p* = 0.0015), FOS‐G (*p* = 0.0013), and GOS‐G (*p* = 0.0001) compared to the base. Notably, the total bacterial abundance was significantly increased by inulin‐G (*p* = 0.0017), FOS‐G (*p* = 0.0003), GOS‐G (*p* = 0.0002), and cacao‐G (*p* = 0.0141) compared to the base. Overall, prebiotic‐containing granola enhanced the abundance of gut microbiota more effectively than granola alone after 48 h of fermentation.

## 4 Discussion

In this study, six different prebiotic materials were added to pig feces and incubated using an in vitro anaerobic fermentation model for 48 h. The prebiotics were metabolized by gut bacteria present in the feces, leading to significantly enhanced SCFA production. In this experimental system, a 24‐h incubation was insufficient to detect clear differences in SCFA formation, whereas 48‐h incubation allowed for more pronounced and consistent observations. This aligns with previous studies using pig fecal models, where SCFA production was reported to increase continuously up to 48 h (Nagata et al. 2021; Sato et al. 2020). However, when examining individual SCFA levels after 24 h, acetic acid concentrations were significantly higher in all treatment groups except the cacao mass group, compared to the control. In contrast, no significant increases in butyric or propionic acid concentrations were observed at that time point. SCFAs are produced at different stages of microbial fermentation (Nagata et al. 2021). Acetic acid can serve as a precursor for butyric acid, while lactic acid can be further metabolized into both butyric and propionic acids. After 24 h, acetic acid is likely produced rapidly, with subsequent conversion into butyric and propionic acids occurring after sufficient accumulation. At 48 h, acetic acid remained the most abundant SCFA, followed by propionic acid, with butyric acid showing the lowest concentrations among the three. Notably, propionic acid levels were markedly lower in the cacao mass group compared to other groups. This suggests that the acetic acid pathway was dominant and that the preferential metabolic pathway may vary depending on the specific prebiotic used. Similar findings have been reported in clinical studies, where the type of SCFA produced differed depending on the type of dietary fiber consumed (Eastwood 1992; Sato et al. 2020). In those studies, acetic acid was typically the most abundant SCFA, followed by propionic and butyric acids—consistent with our results.

Notably, inulin, barley, FOS, and GOS produced comparable total amounts of SCFAs after 48 h of incubation. In contrast, RS produced a slightly lower amount, while cacao mass resulted in a significantly lower level of SCFA production. In this experiment, inulin, FOS, RS, and GOS were added directly to the fermentation system without any prior processing. However, for cacao mass and barley, the indigestible components were extracted using enzymatic digestion, and only the components precipitated with ethanol were collected and used. The barley used in the experiment contained substantial amounts of β‐glucan, fructan, high‐amylose RS, and other indigestible components, similar to those found in other prebiotics such as inulin. In contrast, the main dietary fiber component of cacao mass is lignin (Handojo et al. 2019; McCleary and McLoughlin 2022). Lignin contains various aromatic structures that are resistant to enzymatic degradation and can be broken down by only a limited number of microbial enzymes. Therefore, the lower SCFA production observed for cacao mass may be attributed to its lignin‐rich composition and limited fermentability. In this study, we were unable to analyze the specific components of cacao mass after enzymatic treatment. Thus, further research is needed to investigate the prebiotic potential of cacao mass through detailed compositional analysis before and after digestive processing. This study suggests that prebiotic materials containing indigestible sugar chain–based components promote high SCFA production, whereas dietary fibers rich in lignin are associated with low SCFA production.

Evaluation of total bacterial abundance at 24 h revealed that inulin, barley, FOS, and GOS significantly increased bacterial levels compared to the cellulose control group. In contrast, the RS and cacao mass groups showed no significant difference from cellulose, with minimal increases in bacterial abundance observed for cacao mass. The first four materials (inulin, barley, FOS, and GOS) were either highly water‐soluble or contained a large proportion of water‐soluble components, and thus exhibited high fermentation rates. By contrast, RS and cacao mass (after digestive processing) are composed primarily of insoluble components, resulting in lower fermentation rates and limited fermentability. These compositional differences were clearly reflected in bacterial growth dynamics. Although bacterial abundance plateaued after 24 h in the highly fermentable groups, it continued to increase over 48 h in the RS and cacao mass groups. This gradual fermentation profile may offer physiological benefits by preventing rapid microbial shifts and over‐fermentation in the gut.

The evaluation system used in this study did not clearly identify specific target bacteria for each prebiotic material. For example, although inulin significantly increased the abundance of its target bacterium *Bacteroides* compared to cellulose, it also increased the abundances of *Faecalibacterium*, *Bifidobacterium*, and *Ruminococcus*. Similarly, FOS increased the abundance of its target *Faecalibacterium* more than the other prebiotics, but it also enhanced *Bifidobacterium* and *Ruminococcus* levels. RS increased not only *Bifidobacterium* (its target) but also *Bacteroides*, *Prevotella*, and *Ruminococcus*. GOS significantly increased the abundance of both the target (*Bifidobacterium*) and non‐target (*Faecalibacterium* and *Ruminococcus*) bacteria. Barley significantly increased the abundance of its target bacterium *Prevotella*, as well as *Bacteroides*, *Faecalibacterium*, *Ruminococcus*, and *Bifidobacterium*, indicating a broad stimulatory effect on bacterial growth. In contrast, cacao mass did not significantly increase the abundance of its target bacterium *Blautia*. *Blautia* is a major genus in the gut microbiota of humans, pigs, horses, and dogs, and its optimal growth conditions are near pH 7 and approximately 37°C (Shin et al. 2018; Wang et al. 2021). However, the pH in our culture system was maintained at approximately 5.5, which may not have supported optimal *Blautia* growth. Thus, the system may not have fully revealed the potential of cacao mass to stimulate *Blautia*. Nevertheless, cacao mass significantly increased the abundances of *Bacteroides*, *Prevotella*, *Faecalibacterium*, and *Ruminococcus*, supporting its beneficial effects on gut bacterial growth and its potential as a prebiotic material.

Prebiotic ingredients can be broadly classified based on their effects on target bacterial growth. In this study, the tested ingredients can be grouped into two categories: (inulin, FOS, and GOS) and (barley, RS, and cacao mass). The first group promoted the growth of *Faecalibacterium*, *Ruminococcus*, and *Bifidobacterium*, while the second group favored the proliferation of *Bacteroides* and *Prevotella*. Notably, the first group consisted mainly of water‐soluble fibers, whereas the second contained a variety of insoluble components. In vivo, both soluble and insoluble dietary fibers contribute to SCFA production (Cheng et al. 2017; Sasaki et al. 2020; Zhang et al. 2024). For instance, Cheng et al. (2017) reported that mice fed a 1:1 mixture of soluble and insoluble fibers showed a greater increase in microbial diversity and more pronounced shifts in microbiota composition than those fed soluble fiber alone. Similarly, Sasaki et al. (2020) showed that Jerusalem artichoke powder, which contains both fiber types, enhanced SCFA production more than its water‐soluble extract. Zhang et al. (2024) further demonstrated that the ratio of soluble to insoluble fiber affects SCFA profiles: increasing soluble fiber proportionally increased acetic and propionic acid levels, while butyric acid production peaked at a 6:4 soluble‐to‐insoluble fiber ratio. Insoluble fiber is poorly fermentable and yields minimal metabolites such as SCFAs. However, in vivo, it absorbs water in the stomach and small intestine, swells, and stimulates intestinal peristalsis (Soliman 2019). When combined with soluble fibers, this mechanical property enhances gut motility and contributes to greater SCFA production than soluble fiber alone. Therefore, both the mechanical action of insoluble fibers and the fermentability of soluble fibers are critical factors in SCFA generation. Our in vitro findings also reflected differences in microbial composition depending on fiber solubility. A deeper understanding of how real food—containing a mix of soluble and insoluble fibers—interacts with intestinal peristalsis could provide valuable insight into how diet influences gut microbiota composition and SCFA production. Further research is needed to clarify these mechanisms.

To better understand the fermentation characteristics of each prebiotic ingredient, we conducted tests using processed prebiotic‐containing granolas. All granola samples, including the base granola, were treated with digestive enzymes prior to fermentation. Notably, all granolas produced significantly higher levels of SCFAs than cellulose after both 24 and 48 h of incubation. The overall trends in total SCFA production from prebiotic‐containing granolas were consistent with those observed for the prebiotic ingredients alone: inulin, barley, FOS, and GOS showed significantly high SCFA production, whereas RS and cacao mass showed modest increases. However, changes in bacterial abundance in response to prebiotic‐containing granolas were more complex than those observed with the prebiotic materials alone. For example, *Prevotella* abundance increased only slightly with inulin alone but showed a significant increase when inulin was incorporated into granola. Similarly, while FOS alone did not significantly increase its target bacterium *Prevotella*, FOS‐containing granola did. In the case of *Blautia*, no significant increase in abundance was observed with any prebiotic material alone. However, when inulin, FOS, or GOS was incorporated into granola, *Blautia* abundance increased significantly. This may be due to trace elements or other micronutrients present in the granola that improved the culture environment for *Blautia*, providing more favorable growth conditions than prebiotics alone. These findings suggest that the growth and metabolic activity of gut bacteria are influenced not only by prebiotic substrates but also by food‐derived micronutrients. In some cases, either component may be sufficient, while in others, their combination may be necessary to elicit measurable effects.

In this study, we used base granola and various prebiotic‐containing granolas to examine their effects on SCFA production and gut microbiota. SCFA and bacterial count analyses revealed that base granola alone exhibited high fermentability and promoted bacterial growth to a degree comparable to that of prebiotic‐containing granolas. However, the abundance of target bacteria was significantly higher in the prebiotic‐containing granola groups, particularly after 48 h of incubation. In this in vitro system, prebiotic‐containing granolas altered both the composition and total abundance of the gut microbiota, depending on the type of prebiotic ingredient. While both base and prebiotic‐containing granolas changed the microbiota composition, no significant differences were observed in the total SCFA concentrations. Gut microbiota diversity plays a crucial role in maintaining intestinal homeostasis. When different food components reach the colon, the microbiota can regulate SCFA production to avoid drastic shifts in microbial composition. SCFA production is not determined solely by the prebiotic material added or the specific bacterial taxa present, but rather by complex microbial interactions—including the accumulation of intermediate metabolites such as lactic and succinic acids—prior to SCFA formation (Flint et al. 2012; Louis and Flint 2017). In this study, we focused on major SCFAs (acetic, butyric, and propionic acids), but further investigation of minor SCFAs (e.g., valeric and caproic acids) and SCFA precursors (e.g., lactic and succinic acids) could provide deeper insights into the interactions between prebiotic‐containing granola, SCFA production, and gut microbial ecology. Overall, this study confirmed that prebiotic‐containing granola promotes the growth of multiple bacterial taxa, including target species, and alters microbiota composition. These findings suggest that prebiotic‐containing granolas increase total bacterial abundance more effectively than base granola alone.

## 5 Conclusion

In summary, this study demonstrated the effects of prebiotic‐containing granola on gut microbiota using an in vitro anaerobic fermentation model with pig feces. Each formulation induced distinct changes in the gut microbiota and promoted SCFA production by supporting the growth of both target and non‐target bacterial species. Overall, these findings provide valuable insights into the microbiota‐modulating potential of prebiotic‐containing granola and may inform future human intervention studies.

## Author Contributions

**Hiroyuki Sasaki:** formal analysis (lead), investigation (lead), visualization (lead), writing – original draft (lead). **Hirofumi Masutomi:** conceptualization (equal), project administration (equal), writing – review and editing (equal). **Yota Kobayashi:** formal analysis (equal), investigation (equal), writing – original draft (equal). **Kiyotsuna Toyohara:** project administration (equal), validation (equal), writing – original draft (equal). **Keiichiro Imaizumi:** formal analysis (equal), investigation (equal), validation (equal), writing – review and editing (equal). **Yasunori Nakayama:** conceptualization (equal), project administration (equal), validation (equal), writing – review and editing (equal). **Katsuyuki Ishihara:** supervision (lead), writing – review and editing (equal).

## Ethics Statement

The authors have nothing to report. Pig feces used in this study were obtained from a third party (Obihiro University of Agriculture and Veterinary Medicine (Hokkaido, Japan)), and the authors were not involved in the breeding, sampling, or experimentation of the animals.

## Conflicts of Interest

Hiroyuki Sasaki, Hirofumi Matsumoto, and Katsuyuki Ishihara are employees of Calbee Inc. Yota Kobayashi, Kiyotsuna Toyohara, Keiichiro Imaizumi, and Yasunori Nakayama are employees of Teijin Limited.

## Supporting information


Table S1.

## Data Availability

The data, codebook, and analysis code generated/used in this study are available upon reasonable request from the corresponding author.
