# Supplementary material for: Evaluation of the Fermentation Characteristics of Prebiotic‐Containing Granola and Short‐Chain Fatty Acid Production in an In Vitro Gut Microbiota Model
Source: Food Sci Nutr. 2025 May 7;13(5):e70252. doi: 10.1002/fsn3.70252 (PMC12058461; doi:10.1002/fsn3.70252)
Supplement: Supplementary file 1 — Table S1. [file FSN3-13-e70252-s001.docx]

Supplemental Table 1. Ingredient composition of each granola containing prebiotics.

| Base | Inulin-G | Barley-G | FOS-G | RS-G | GOS-G | Cacao-G |
| --- | --- | --- | --- | --- | --- | --- |
| Oats, dried fruit (papaya, raisins, apples, strawberries), rye flour, sugar, wheat flour, coconut, dextrin, vegetable oil, rice flour, corn flour, soluble dietary fiber, pumpkin seeds, almond powder, salt, wheat bran , brown rice flour, fructooligosaccharide syrup, lactose / glycerin, sodium citrate, acidulant, antioxidants (vitamin E, rosemary extract), modified starch, niacin, calcium pantothenate, vitamin A, vitamin B6, vitamin B1, folic acid, vitamin D, vitamin B12 | Oats, rice puffs, inulin, wheat puffs (wheat flour, starch, malt extract, salt), sugar, dextrin, almond powder, salt / flavoring, leavening agent, antioxidant (vitamin E) | Oats, rice puffs, barley, wheat puffs (wheat flour, starch, malt extract, salt), dextrin, sugar, almond powder, salt / leavening agent, antioxidant (vitamin E) | Oats, wheat puffs (wheat flour, starch, malt extract, salt), fructo-oligosaccharides, wheat flour, dextrin, rice flour, corn flour, rye flour, almond powder, salt, brown rice flour / flavoring, leavening agent, antioxidant (vitamin E) | Resistant starch, wheat flour, sugar, vegetable oil, rice flour, corn flour, rye flour, brown rice flour, salt, dextrin / flavoring, safflower yellow, antioxidant (vitamin E) | Rice puffs, galacto-oligosaccharide (contains milk ingredients), oats, wheat flour, dextrin, rice flour, corn flour, rye flour, almond powder, salt, brown rice flour / flavoring, antioxidant (vitamin E) | Wheat flour, sugar, oats, chocolate (contains milk ingredients), wheat puffs (wheat flour, starch, malt extract, salt), rice flour, corn flour, cacao mass, rye flour, almond powder, dextrin, brown rice flour, salt / emulsifier (soybean derived), leavening agent, antioxidant (vitamin E), flavoring |

Base: Base granola, Inulin-G: Granola containing inulin, Barley-G: Granola containing barley, FOS-G: Granola containing Fructo-Oligosaccharides, RS-G: Granola containing Resistant Starch, GOS: Granola containing Galacto-oligosaccharides. Cacao-G: Granola containing cacao mass.
